# A case of secretory azoospermia: is valganciclovir responsible?

**DOI:** 10.1186/s12610-026-00299-0

**Published:** 2026-07-06

**Authors:** Louise Michenaud, Marion Lapoirie, Eloïse Fraison, Bruno Salle-Leyris-de-Campredon, Ingrid Plotton, Sandrine Giscard-d’Estaing

**Affiliations:** 1 Service de Médecine de la Reproduction et Préservation de la Fertilité, Hôpital Femme Mère Enfant, Hospices Civils de Lyon, HFME, 59, Boulevard Pinel, Bron, 69500 France; 2 Inserm U1208, Bron, France; 3 Université Claude Bernard Lyon I, 43 Boulevard du 11 Novembre 1918, Villeurbanne, 69100 France

**Keywords:** Reprotoxicity, Azoospermia, Iatrogenic, Ganciclovir, Spermatogenesis, Transplant, Hyperthermia

## Abstract

**Background:**

Valganciclovir, the prodrug of ganciclovir, is an antiviral agent targeting herpes viruses. It is indicated for the prophylaxis or treatment of cytomegalovirus infections in immunocompromised patients. Pre-clinical studies state ganciclovir as a mutagenic, teratogenic, reprotoxic drug that can rarely alter male fertility.

**Case presentation:**

Mr S. had experienced primo-secondary infertility with his partner for 12 months. He had no significant medical history aside from exertional asthma and achieved semen analysis at age 33, revealing normal sperm concentration (110 million/mL, 383 million/ejaculate) with normal progressive motility (50%).

One month after, during a trail race, he reported malignant exertional hyperthermia (41.7°C) leading to multi-organ failure and coma and requiring intensive care and an emergency hepatic transplant. Post-transplant, anti-rejection therapy was started including tacrolimus, mycophenolate mofetil, co-trimoxazole, and valganciclovir.

Six months after the transplant, he undertook sperm cryopreservation. Repeated semen analysis confirmed azoospermia.

Semen analysis 12 weeks after discontinuation of valganciclovir showed rare motile sperm, allowing cryopreservation. Subsequent analyses showed significant improvement, with concentration reaching 10.1 M/mL 14 weeks later and 70 M/mL 24 weeks later, allowing ICSI with fresh sperm. This resulted in a successful pregnancy and live birth.

**Conclusions:**

We report a case of azoospermia observed after valganciclovir treatment. Based on a review of the literature, the azoospermia observed in this patient could be attributed to valganciclovir.

However, alternative explanations such as fever or infectious complications cannot be ruled out. Valganciclovir is reprotoxic and should be replaced with an alternative drug in men planning to conceive.

## Résumé

**Contexte:**

Le valganciclovir, le promédicament du ganciclovir, est un agent antiviral ciblant les virus de l’herpès. Il est indiqué dans la prophylaxie ou le traitement des infections à cytomégalovirus chez les patients immunodéprimés. Les études précliniques indiquent que le ganciclovir est un médicament mutagénique, tératogène et reprotoxique, qui peut rarement altérer la fertilité masculine.

**Présentation du Cas:**

Mr S. avait vécu une infertilité primo-secondaire avec sa partenaire pendant 12 mois. Il n’avait pas d’antécédents médicaux significatifs à part un asthme d’effort ; il a réalisé une analyse de sperme à 33 ans, qui révélait une concentration normale de spermatozoïdes (110 millions/mL ; 383 millions/éjaculat) avec une motilité progressive normale (50 %).

Un mois plus tard, lors d’une course de trail, il a signalé une hyperthermie d’effort maligne (41,7°C) entraînant une défaillance multi-organes et un coma, qui nécessitât des soins intensifs et une greffe hépatique d’urgence. Après la greffe, une thérapie antirejet a été commencée, incluant le tacrolimus, le mycophénolate mofetil, le co-trimoxazole et le valganciclovir.

Six mois après la greffe, il entreprit une cryopréservation du sperme. Des analyses répétées de sperme confirmèrent la présence d’une azoospermie.

Une analyse du sperme 12 semaines après l’arrêt du valganciclovir a révélé de rares spermatozoïdes mobiles, permettant une cryopréservation. Les analyses ultérieures ont montré une amélioration significative, avec une concentration atteignant 10,1 M/mL 14 semaines plus tard, et 70 M/mL 24 semaines plus tard, permettant une ICSI avec des spermatozoïdes frais. Ceci aboutit à une grossesse réussie et à une naissance vivante.

**Conclusions:**

Nous rapportons un cas d’azoospermie observée après un traitement au valganciclovir. Après revue de la littérature, l’azoospermie observée chez ce patient pourrait être attribuée au valganciclovir. Cependant, d’autres explications, telles que la fièvre ou les complications infectieuses, ne peuvent être exclues. Le valganciclovir est reprotoxique et doit être remplacé par un médicament alternatif chez les hommes envisageant de concevoir.

## Background

Valganciclovir (VGCV) is the prodrug of ganciclovir (GCV): after oral administration, VGCV is extensively metabolized to GCV by intestinal and hepatic esterases [1]. GCV is an antiviral agent that targets Herpesviridae by inhibiting their replication. GCV acts preferentially upon virus-infected cells in its triphosphorylated state. The phosphorylation of GCV by the viral kinase is a key step, to obtain the active form. In its triphosphorylated state, GCV is an analogue of deoxyguanosine triphosphate and competitively inhibits its incorporation into deoxyribonucleic acid (DNA) by viral DNA polymerase. Thus, incorporation into viral DNA leads to termination of elongation.

This drug is required in the prophylaxis or treatment of cytomegalovirus infections in immunocompromised patients such as those who have undergone organ transplants or have AIDS. During treatment, the patient’s blood cell counts and platelet levels must be monitored due to the risk of cytopenia or even bone marrow aplasia. Because GCV-triphosphate is incorporated into DNA and inhibits DNA polymerases, it affects rapidly dividing human cells such as hematopoietic cells, but also germ cells, thereby explaining the potential to induce (0,1 to 1%) reproductive toxicity, mutagenicity and teratogenicity [2–4].

We report the follow up of a patient who developed azoospermia following treatment with VGCV.

## Case presentation

Mr S. ha experienced primo-secondary infertility with his partner for 12 months. He had no significant medical history aside from exertional asthma. The couple faced an ectopic pregnancy treated with methotrexate in June 2022, followed by an early miscarriage at six weeks of amenorrhea.

His partner’s infertility evaluation, at age 27, revealed an Anti-Mullerian Hormone level of 1.5 ng/mL, patent fallopian tubes and deep endometriosis affecting the torus and the two utero-sacral ligaments, along with bilateral endometriomas.

**Fig. 1:**
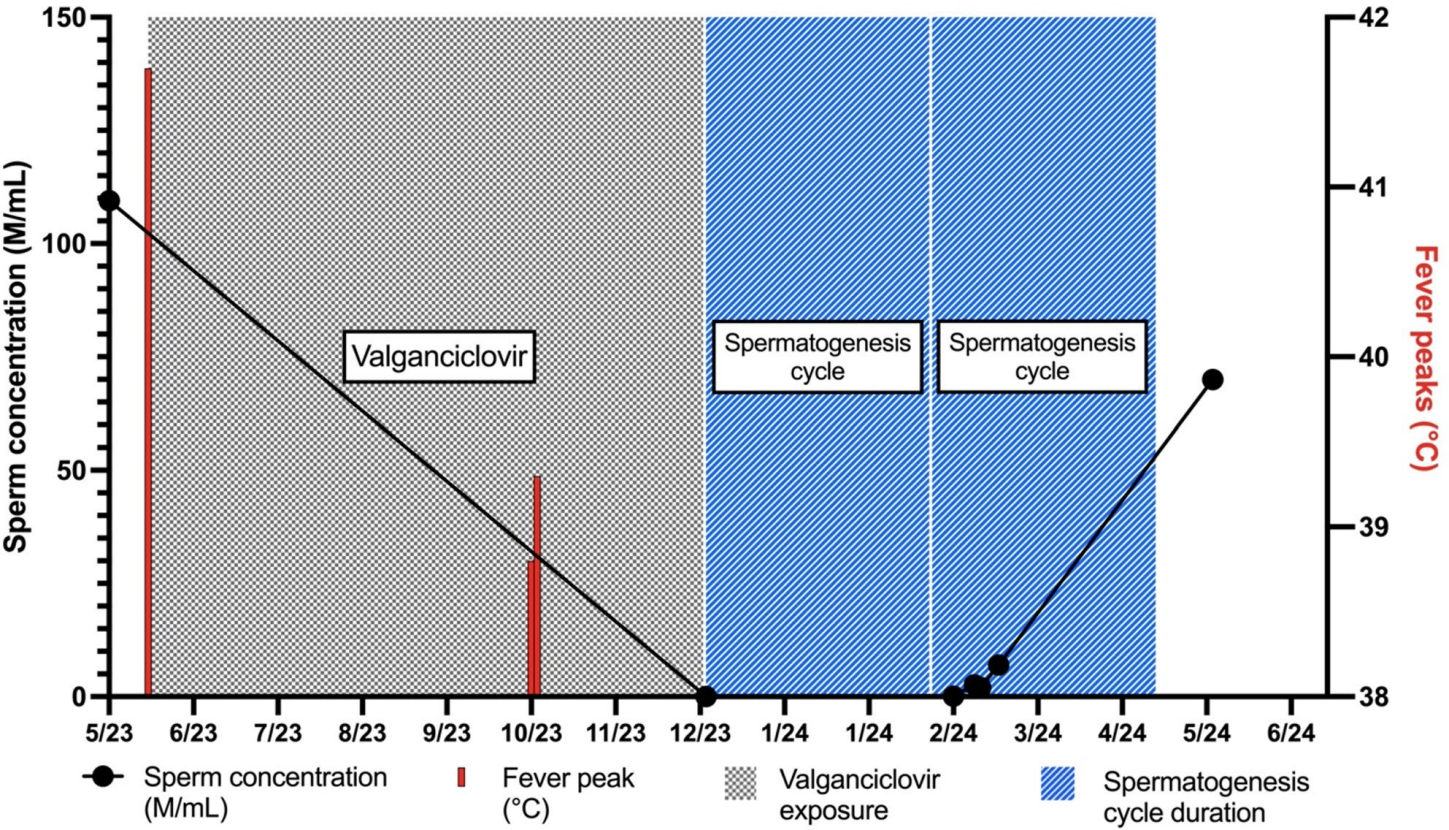
Evaluation of sperm concentration (M/mL) over time, including fever peaks (°C), VGCV treatment and duration of a spermatogenesis cycle (74 days)

At age 33, he underwent a semen analysis that revealed satisfactory sperm concentration (110 million/mL, World Health Organization WHO normality threshold ≥ 16 million/mL [5], 383 million/ejaculate (Table 1; Fig. 1), normal progressive motility (50%, WHO normality threshold ≥ 30%), moderate isolated teratozoospermia (18% typical forms according to the modified David classification, internal laboratory threshold ≥ 20%), and a migration-survival test compatible with insemination (53 million progressive typical spermatozoa per ejaculate after treatment).

**Table 1:** Chronology of key events in the patient’s case

Date	Event
May 2023	Semen analysis: subnormal
3 June 2023	Trail race
6 June 2023	Hepatic transplant
September 2023	Therapeutic window for sperm cryopreservation: discontinuation of mycophenolate mofetil and cotrimoxazole
October 2023	Two febrile peaks
December 2023	Semen analysis: azoospermia; discontinuation of valganciclovir
February 2024	Semen analysis: 2.6 M/mL; beginning of cryopreservation process
March 2024	Semen analysis: 10.1 M/mL
May 2024	ICSI: 70 M/mL
January 2025	Birth of a healthy baby boy

Dates correspond to the timing of treatments, laboratory tests, and procedures throughout the course of follow-up

One month later, Mr. S. participated in a trail race and subsequently suffered from malignant exertional hyperthermia (41.7 °C) associated with multi-organ failure and coma. He was admitted to intensive care unit, required an emergency hepatic transplant 3 days after the race, and was intubated for 7 days (Table 1). He also experienced rhabdomyolysis, renal failure treated with dialysis (with partial recovery), disseminated intravascular coagulation with thrombosis of the external iliac and left common femoral veins treated with heparin, resuscitation neuromyopathy, and suspected prostatitis (*E. coli*) treated with piperacillin-tazobactam. Post-transplant, he was started on anti-rejection medications including tacrolimus, mycophenolate mofetil, co-trimoxazole, and VGCV.

Mycophenolate mofetil, known to be mildly clastogenic, is recommended to be suspended for a spermatogenesis cycle (three months) in a pre-conception setting. There is no data on the reproductive toxicity of cotrimoxazole in men, but it is recommended to be suspended or replaced in pregnant women due to its teratogenicity. Three months later, the decision was made to temporarily stop mycophenolate mofetil and co-trimoxazole (the latter as a precautionary measure) to initiate sperm freezing and continue the current parental project. In December 2023, six months post-hospitalization, Mr. S. visited our Center for the Study and Preservation of Human Eggs and Semen to begin the sperm cryopreservation process. He had experienced two febrile peaks on October 11th and 13th (38.8 °C and 39.3 °C, respectively) with no identified etiology (Table 1). He had been free of toxics since hospitalization and was being treated with azathioprine, tacrolimus, atovaquone, aspirin, amlodipine and VGCV.

The semen analysis conducted after a four-day period of abstinence showed no spermatozoa on direct examination, even after thorough examination of the entire centrifugation pellet (Table 1; Fig. 1). The semen pH was normal, and the sample contained 1 million/mL round cells, 58% of which were leukocytes. An andrological consultation, a karyotype analysis, a testicular ultrasound, and hormonal blood works were prescribed, with a follow-up appointment scheduled for three months later.

The hormonal evaluation mentioned spermatogenesis failure with elevated follicle-stimulating hormone (FSH) and luteinizing hormone (LH) levels (respectively 11 and 9 IU/L), slightly increased prolactin (22 µg/L), total testosterone in upper range (31 nmol/L), associated with elevated sex hormone-binding globulin (88 nmol/L, normal values 20.9–50.6 nmol/L). Bioavailable testosterone was diminished at 1.1 nmol/L (normal values 2.25–10.7 nmol/L), estradiol was normal (297 pmol/L), and inhibin B was decreased at 9 ng/L. The karyotype was normal (46, XY) and the testicular ultrasound showed no abnormalities.

Three months after, Mr. S. was re-evaluated by the andrologist after completing rehabilitation and resuming physical activity. At this time, he was being treated with tacrolimus, atovaquone, aspirin, and amlodipine, while VGCV was discontinued in mid-December 2023, after his visit in our center (Table 1; Fig. 1), after 6 months of treatment, according to international consensus guidelines [6].

The potential causes of azoospermia included VGCV toxicity, prostatitis-induced orchiepididymitis, testicular distress during multi-organ failure episode (explaining the hormonal imbalance), hyperthermia, or a combination of these factors.

14 weeks after discontinuing VGCV, a new semen analysis showed the resumption of spermatogenesis (2.6 million/mL), and cryopreservation was initiated. The hormonal evaluation showed normal levels of adrenocorticotropic hormone (ACTH), growth hormone (GH), insulin-like growth factor 1 (IGF-1), prolactin, thyroid-stimulating hormone (TSH), T4, and T3. FSH, LH, and estradiol levels were also normal. Inhibin B and bioavailable testosterone levels had increased since December but remained low (34 ng/L and 1.5 nmol/L, respectively, Table 1; Fig. 1).

Throughout, the patient’s sperm characteristics improved significantly, reaching a concentration of 10.1 M/mL, and then 70 M/mL 24 weeks after discontinuation (Table 1; Fig. 1).

Overall, 20 sperm straws were cryopreserved in our center.

Following advice from the French Reference Center on Teratogenic Agents, the semen straws preserved during the 3-month period after discontinuation of VGCV were set aside because of VGCV’s genotoxicity. By May 2024, 6 months after VGCV discontinuation, the couple proceeded with their first intracytoplasmic sperm injection (ICSI) procedure (Table 1; Fig. 1). Since fresh sperm characteristics had significantly improved, almost back to pre-transplant state, with a concentration of 70 M/mL, 45% progressive motility, and 20% typical forms, the fresh sample was utilized instead of preserved semen straws. Even if semen parameters were normal, ICSI strategy was maintained, as discussed and consented by the couple. One blastocyst was transferred at day five, resulting in pregnancy and the birth of a healthy baby boy in January 2025. Eight supernumerary blastocysts were frozen at day five and day six.

The patient provided written informed consent to participate in this case report, and approval was obtained from the institutional ethics committee (authorization no. 25–357).

## Discussion and conclusions

We report the first case of azoospermia following valganciclovir therapy initiated after liver transplantation, and the second case of transient azoospermia observed after valganciclovir treatment post-transplant [7]. This is also the first published instance of a live birth following valganciclovir-related azoospermia.

Post hepatic transplantation immunosuppressive therapy typically involves a combination of calcineurin inhibitors (e.g., tacrolimus), antimetabolites (e.g., mycophenolate mofetil), and corticosteroids. This regimen aims to prevent acute rejection while minimizing the risk of chronic graft dysfunction. Prophylactic strategies against infections in liver transplant recipients include the use of antiviral agents to prevent cytomegalovirus (CMV) infection, antifungal agents to prevent fungal infections, and antibacterial agents to prevent bacterial infections. The duration and choice of prophylaxis are tailored based on individual patient risk factors and institutional protocols.

The literature overview about etiologies of this azoospermia was carried out and particularly focused on the treatments prescribed for the patient after his transplant. We then used scores to determine the causality of VGCV in his azoospermia (Table 2).

**Table 2:** Summary of literature on reproductive toxicity of treatments administered to the patient

Treatment	Class	In vitro	In vivo
Tacrolimus	Immunosuppressant	Mutagenic, cytotoxic [8–10]	No adverse effects attributable to paternal treatment (limited data) [11]
Mycophenolate	Immunosuppressant	Clastogenic, mutagenic, genotoxic [8, 12, 13]	No adverse effects attributable to paternal treatment (limited data) [11]
Cotrimoxazole	Antibiotic, antiprotozoal		Teratogenic in women. No published data on paternal treatment. [14, 15]
Azathioprine	Immunosuppressant	Mutagenic, carcinogenic [16]	No adverse effects attributable to maternal or paternal treatments [17]
Atovaquone	Antiprotozoal	Cytotoxic, genotoxic, embryotoxic [18, 19]	No published data on paternal treatment
Aspirin	Nonsteroidal anti-inflammatory		No adverse effects attributable to paternal treatment [16]
Amlodipine	Antihypertensive		No adverse effects attributable to paternal treatment (limited data) [20]
Valganciclovir	Antiviral	Mutagenic, genotoxic, carcinogenic, clastogenic, teratogenic [21–23]	Temporary inhibition of spermatogenesis and genotoxic damages [3, 7, 24–28] No data on health of children conceived under paternal treatment

### Reprotoxicity of tacrolimus

Tacrolimus, a calcineurin inhibitor immunosuppressant, is indicated for the prevention of allograft rejection in organ transplantation and for the treatment of certain immune-mediated inflammatory disorders. In preclinical testing, tacrolimus showed no mutagenic or clastogenic effects [29]. It is not teratogenic when used in maternal treatment during pregnancy. However, a few studies have shown mutagenic and cytotoxic effects on human cells [8–10]. There are limited data on children conceived by men undergoing tacrolimus treatment (40 pregnancies), but no adverse effects attributable to paternal treatment have been reported to date. Based on this information, fertility preservation and conception are possible with tacrolimus during paternal treatment [11].

### Reprotoxicity of mycophenolate

Mycophenolate mofetil, an antimetabolite immunosuppressant, is indicated for the prevention of allograft rejection in organ transplantation and for the treatment of selected immune-mediated disorders. Mycophenolate is mildly clastogenic in experimental studies [12]. Some studies have also demonstrated genotoxicity in the spleen and bone marrow of rats, enhanced by association with tacrolimus, as well as mutagenicity on human lymphocyte cultures [8, 30]. While many pregnancies have been successfully carried out with the father undergoing mycophenolate treatment, no adverse effects attributable to paternal treatment have been reported to date, in contrast to the effects observed with maternal treatment. As a precautionary measure and pending further data, it is recommended to suspend or replace mycophenolate for at least one spermatogenesis cycle (three months) before conception, if permitted by the patient’s clinical situation [13].

### Reprotoxicity of cotrimoxazole

Cotrimoxazole, a fixed-dose combination of the antimicrobial agents sulfamethoxazole and trimethoprim, is indicated for the treatment and prophylaxis of bacterial and opportunistic infections, including *Pneumocystis jirovecii* pneumonia. Cotrimoxazole teratogenicity has been established in pregnant women, with a doubling of the overall frequency of malformations compared with the general population [14]. This may be due to the anti-folate action of trimethoprim, associated with an increased risk of neural tube defects. According to the French Teratogen Information Centre, during the first 10 weeks of amenorrhea, cotrimoxazole should be avoided or replaced if possible [15]. No data suggesting reprotoxicity in male has been published.

### Reprotoxicity of azathioprine

Azathioprine, an antimetabolite immunosuppressant, is indicated for the prevention of allograft rejection in organ transplantation and for the management of various autoimmune and inflammatory disorders. Although azathioprine exhibits mutagenic and carcinogenic properties in vitro, extensive published data on pregnant women exposed during the first trimester and their offspring are abundant and reassuring [16]. Paternal exposure does not appear to significantly affect male fertility or increase the risk of birth defects in offspring [17].

### Reprotoxicity of Atovaquone

Atovaquone is an antiparasitic and antiprotozoal agent that specifically and potently inhibits the mitochondrial electron transport chain. It is indicated for the prevention of *Pneumocystis jirovecii* infections in transplant patients. In preclinical safety tests, atovaquone has been shown to have a weak cyto-genotoxic potential in vitro [18]. In vivo, studies on rabbits revealed maternal and embryotoxic effects at high doses, but no impairment of fertility has been described in animals [19]. To date, there are no published data on the fertility of men treated with atovaquone or on pregnancies conceived by men undergoing this treatment. According to available data, fertility preservation and conception are possible with atovaquone during paternal treatment.

### Reprotoxicity of aspirin and amlodipine

Aspirin, a nonsteroidal anti-inflammatory drug (NSAID) and antiplatelet agent, is indicated for analgesia, antipyresis, anti-inflammatory therapy, and the prevention of cardiovascular events such as myocardial infarction and stroke. Amlodipine, a dihydropyridine calcium channel blocker, is indicated for the treatment of hypertension and *angina pectoris*. Paternal exposure to low-dose aspirin has not been associated with adverse effects on male fertility or increased risk of birth defects in offspring [16]. Similarly, limited data indicate that paternal amlodipine exposure does not appear to compromise sperm quality or reproductive outcomes [20].

### Reprotoxicity of VGCV

Ganciclovir, a nucleoside analogue antiviral, is indicated for the treatment and prevention of cytomegalovirus (CMV) infections in immunocompromised patients, particularly transplant recipients and individuals with advanced HIV infection. GCV is well-known for its mutagenic, genotoxic, carcinogenic, and clastogenic properties [21–23]. It is also teratogenic in animals, causing embryo lethality and fetal growth retardation, which counter-indicates its use in pregnancy despite its effectiveness in preventing congenital cytomegalovirus (CMV) infection. A 1999 study found no teratogenic effects on babies born to female transplant recipients who received GCV during their pregnancy [31]. Recently, a 2021 review reported seven cases of GCV or VGCV use during pregnancy for fetal or maternal CMV infection, with no reported negative effects on the fetus [32]. A pharmacovigilance study published in 2023 showed no increase reporting of any adverse pregnancy outcome or birth defects with GCV compared with acyclovir during pregnancy. Nonetheless, four cases of esophageal and anorectal atresia were identified in the GCV cohort, potentially related to concomitant medications or underlying medical conditions. These findings suggest that GCV may be considered a relatively safe treatment for congenital CMV infection in both mother and fetus, considering the risk-benefit balance. CMV infection is the leading cause for sensorineural hearing loss and development delays in children without genetic diseases worldwide. It suggests the possibility for trial evaluation of GCV in severe maternal or fetal CMV infections [4, 33, 34].

In preclinical studies, GCV has been shown to inhibit spermatogenesis but long-term effects on fertility are unknown [24]. In 1995, Neyts et al. showed that GCV caused atrophy of testicular germinal epithelium in mice with no spermatozoa in the epididymal channels, while Sertoli cells were preserved [25]. In 1997, Faqi et al. showed that GCV caused a significant reduction of viable fetuses in rats, as well as a reversible reduction of daily sperm production and sperm count, below therapeutic levels. Damages were mostly noticed in the early stages of spermatogenesis. Sertoli cells were swollen and abnormal sperm morphology was described. GCV effects were reversible partially 12 weeks after stopping treatment, and totally 24 weeks after stopping treatment [3].

The only human trial about GCV’s spermatotoxicity was conducted by McLeroth et al. in 2020. They investigated the effects of a 200-day administration of prophylactic doses of GCV (900 mg/day) in 38 male renal transplant recipients. This prospective, multicenter, open-label, non-randomized study assessed semen parameters and DNA fragmentation in patients treated either with GCV or without. In the GCV group, semen parameters showed delayed improvement post-transplant. Six months post-treatment, parameters were comparable between cohorts. There was no significant difference between the two cohorts for sperm DNA fragmentation. The fall in LH was pronounced in the VGCV cohort from baseline to end of treatment and from baseline to end of follow-up. The other parameters evaluated (age, race, duration of dialysis, sperm count) did not influence the outcome [26]. A systematic review of the spermatotoxic and genotoxic effects of multiple anti-rejection drugs published in 2021 concluded that animal and human studies indicate temporary inhibition of spermatogenesis and genotoxic damages by GCV, but no human clinical studies have yet investigated the potential of GCV to damage sperm DNA [27]. A recent case report confirmed this by describing transient azoospermia following treatment with valganciclovir after a kidney transplant. Another study reported one case of azoospermia secondary to treatment for cytomegalovirus uveitis in a cohort of 40 patients treated [7, 28]. As of now, there is no published data on the health outcomes of children conceived by men treated with GCV.

### Causality of VGCV in azoospermia

Causality was assessed using two methods. The French method, as described by Bégaud et al. in 1985 [35], evaluates extrinsic and intrinsic causality, the latter combining chronological causality and semiological causality [35]. In this case, there was likely chronological causality and plausible semiological causality, resulting in a likely intrinsic causality. Extrinsic causality was deemed notable (Table 3).

**Table 3:** Scores for adverse drug-reaction causality by the French method for the patient, adapted from Bégaud et al., 1985 [35] Scores corresponding to the patient’s case are in bold

**Chronological score**		
Time to onset of the adverse reaction	Highly suggestive	C3: likely **C2: plausible** C1: unlikely C0: excluded
	Incompatible	
	**Compatible**	
Dechallenge: evolution after drug withdrawal	**Suggestive**	
	**Non conclusive**	
	**Non suggestive**	
Rechallenge: readministration of the drug	**Positive**	
	**Not realized**	
	**Negative**	
Semiological score		
Evocative of the role of the medication or well-established predisposing factor for the adverse reaction-drug pair	**Evocative of the role of this drug**	S3: likely **S2: plausible** S1: uncertain
	Other situations	
Reliable specific complementary examination or lab test or response to a specific antidote	Positive	
	**Not realized**	
	Negative	
Other non-drug cause	**Present**	
	Absent	
**Intrinsic causality score (chronological + semiological)**	**C2S2**	I4: very likely I3: likely **I2: plausible** I1: doubtful I0: excluded
**Extrinsic or bibliographic score**		
Referenced or reported in reference books or bibliographic databases	**B3: Described or notable**	
	**B2: Rarely described or for close compounds**	
	B1: Unreported effect	
	B0: Exhaustive literature search negative	

The Naranjo method gave a causality score of possible (score = 4) [36]. (Table 4)

**Table 4:** Score for adverse drug-reaction causality by the Naranjo method for the patient, adapted from Naranjo et al., 1981 [36]

	Yes	No	Do not know	Score for the patient’s case
Are there previous conclusive reports on this reaction?	+ 1	0	0	1
Did the adverse event appear after the suspected drug was administered?	+ 2	-1	0	3
Did the adverse reaction improve when the drug was discontinued or a specific antagonist was administered?	+ 1	0	0	4
Did the adverse reaction reappear when the drug was readministered?	+ 2	-1	0	4
Are there alternative causes (other than the drug) that could on their own have caused the reaction?	-1	+ 2	0	3
Did the reaction reappear when a placebo was given?	-1	+ 1	0	3
Was the drug detected in the blood (or other fluids) in concentrations known to be toxic?	+ 1	0	0	3
Was the reaction more severe when the dose was increased, or less severe when the dose was decreased?	+ 1	0	0	3
Did the patient have a similar reaction to the same or similar drugs in any previous exposure?	+ 1	0	0	3
Was the adverse event confirmed by any objective evidence?	+ 1	0	0	4
Total score	4			
≥ 9: definite / 5–8: probable / 1–4: possible / ≤ 0: doubtful				

Although the recovery of spermatogenesis temporally coincided with a full spermatogenic cycle following the discontinuation of VGCV, causality cannot be definitively established and was evaluated plausible with the two causality scores. The patient experienced several acute events known to impair spermatogenesis, including febrile episodes in October 2023, hyperthermia, multi-organ (hepatic) failure, and prostatitis. Hyperthermia and systemic inflammation can disrupt spermatogenesis through germ cell apoptosis and impaired Sertoli cell function. Hepatic dysfunction may alter the hormonal environment critical for normal spermatogenesis. Prostatitis can also negatively impact sperm quality. Semen parameters were not assessed exactly three months after the febrile episodes, while VGCV treatment was ongoing, so a contribution of fever impairment cannot be entirely excluded. Nevertheless, spermatogenesis resumed after the duration of one spermatogenic cycle after VGCV discontinuation, whereas the last fever spikes preceded this period, suggesting a temporal association between VGCV exposure and the observed azoospermia. Taken together, while multiple overlapping factors may have contributed, the timing supports a plausible role of VGCV in transient spermatogenic alteration, although this interpretation must be considered with caution.

### Perspectives

To the best of our knowledge, this case report represents the first documented instance of azoospermia in a man treated with VGCV after a liver transplant. Given the increasing number of patients receiving VGCV, especially transplant recipients, its potential reproductive toxicity should be considered. For men of reproductive age, referral to a sperm and egg preservation center for sperm cryopreservation and fertility counseling should be discussed prior to treatment. In addition, alternative prophylactic strategies exist and may warrant consideration. According to international consensus guidelines, high-dose valaciclovir is effective for CMV prevention in kidney transplant recipients and is associated with less myelotoxicity than oral ganciclovir [6]. Letermovir, a viral terminase complex inhibitor, has also demonstrated efficacy as primary prophylaxis in liver transplant recipients, with reduced need for granulocyte colony-stimulating factor [6]. These agents appear to be less myelotoxic than ganciclovir and could represent potential alternatives, assuming that spermatotoxicity shares mechanistic pathways with myelotoxicity. Prospective clinical studies are needed to evaluate their comparative reproductive safety and to better guide CMV prophylaxis in men of reproductive age. Further studies on the reproductive toxicity of GCV and VGCV, as well as the safety of children born to fathers exposed to these drugs, are warranted.

## Data Availability

No datasets were generated or analysed during the current study.

## Abbreviations

ACTH: Adrenocorticotropin
AIDS: Acquired Immune Deficiency Syndrome
CMV: Cytomegalovirus
DNA: DeoxyriboNucleic Acid
FSH: Follicle-Stimulating Hormone
GCV: Ganciclovir
GH: Growth Hormone
ICSI: IntraCytoplasmic Sperm Injection
IGF-1: Insulin-like Growth Factor 1
LH: Luteinizing Hormone
TSH: Thyroid-Stimulating Hormone
VGCV: Valganciclovir
WHO: World Health Organization
